# Succinate induces aberrant mitochondrial fission in cardiomyocytes through GPR91 signaling

**DOI:** 10.1038/s41419-018-0708-5

**Published:** 2018-06-04

**Authors:** Yi-Tong Lu, Lan-Zhu Li, Yi-Lin Yang, Xiaojian Yin, Qun Liu, Lei Zhang, Kang Liu, Baolin Liu, Jia Li, Lian-Wen Qi

**Affiliations:** 1 State Key Laboratory of Natural Medicines, China Pharmaceutical University, Nanjing, China; 2 Jiangsu Key Laboratory of TCM Evaluation and Translational Research, Department of Pharmacology of Chinese Materia Medica, China Pharmaceutical University, Nanjing, China

## Abstract

Altered mitochondrial metabolism acts as an initial cause for cardiovascular diseases and metabolic intermediate succinate emerges as a mediator of mitochondrial dysfunction. This work aims to investigate whether or not extracellular succinate accumulation and its targeted G protein-coupled receptor-91 (GPR91) activation induce cardiac injury through mitochondrial impairment. The results showed that extracellular succinate promoted the translocation of dynamin-related protein 1 (Drp1) to mitochondria via protein kinase Cδ (PKCδ) activation, and induced mitochondrial fission factor (MFF) phosphorylation via extracellular signal-regulated kinases-1/2 (ERK1/2) activation in a GPR91-dependent manner. As a result, enhanced localization of MFF and Drp1 in mitochondria promoted mitochondrial fission, leading to mitochondrial dysfunction and cardiomyocyte apoptosis. We further showed that inhibition of succinate release and GPR91 signaling ameliorated oxygen–glucose deprivation-induced injury in cardiomyocytes and isoproterenol-induced myocardial ischemia injury in mice. Taken together, these results showed that in response to cardiac ischemia, succinate release activated GPR91 and induced mitochondrial fission via regulation of PKCδ and ERK1/2 signaling branches. These findings suggest that inhibition of extracellular succinate-mediated GPR91 activation might be a potential therapeutic strategy for protecting cardiomyocytes from ischemic injury.

## Introduction

In cardiomyocytes, mitochondrial homeostasis plays a key role in maintaining heart function in response to metabolic stress^1^. Although inflammation, oxidative stress and endoplasmic reticulum stress are involved in cardiac injury, accumulating evidence demonstrates that mitochondrial dysfunction is an initial cause for these events^2,3^. Alterations in mitochondrial morphology increases the susceptibility of the heart to ischemia/reperfusion injury^4,5^, indicating the important role of mitochondrial integrity in the protection of cardiac function.

Mitochondrial morphology is dynamically controlled by continuous fission and fusion. Dynamin-related protein 1 (Drp1) is a central regulator in mitochondrial fission. Drp1 is primarily located in the cytosol. Upon activation, Drp1 is recruited from the cytoplasm to the mitochondrial outer membrane, where it binds to mitochondrial fission factor (MFF) to trigger mitochondrial fission^6^. Drp1 activation is regulated by phosphorylation modification. Protein kinase Cδ (PKCδ) and extracellular signal-regulated kinase-2 (ERK2) are shown to increase Drp1 translocation to mitochondria and promote mitochondrial fission by phosphorylation of Drp1 at Serine 616^7,8^. Moreover, phosphorylation of MFF may regulate the association of Drp1 with mitochondria. This concept is supported by the enhanced binding of Drp1 to MFF when MFF is phosphorylated by AMP-activated protein kinase (AMPK)^9^.

Succinate is an important metabolic intermediate in the citric acid cycle and emerging evidence demonstrates that dysregulation of succinate generation is involved in cardiovascular diseases and metabolic disorders^10^. Although succinate is normally produced in mitochondria, the accumulated succinate can be released to extracellular space in response to pathological status, such as ischemia, diabetes and hypertension^11,12^. It has been demonstrated that extracellular succinate exerts a paracrine and endocrine effector through activation of its specific G protein-coupled receptor-91 (GPR91)^10^. Signaling pathways triggered by GPR91 include activation of PKC and ERK1/2^13,14^. GPR91 is highly expressed in the heart^15^, mediating succinate-induced cardiomyocyte death^16^. Moreover, upholding levels of serum succinate can cause cardiac hypertrophy through activation of GPR91^17^. Given the important role of mitochondrial function in cardioprotection^2,3^, this work aims to investigate whether or not extracellular succinate accumulation and its targeted GPR91 receptor activation induce cardiac injury through mitochondrial impairment.

## Materials and methods

### Reagents and antibodies

Sodium succinate dibasic hexahydrate, dimethyl malonate, rottlerin and tetramethylrhodamine ethyl ester perchlorate (TMRE) were purchased from Sigma-Aldrich (St Louis, MO, USA). Mito Tracker Red CMXRos (M7512) was obtained from Molecular Probes (Thermo Fisher Scientific, San Jose, CA, USA). Cell Tracker CM-Dil and dihydroethidium (DHE) were purchased from Beyotime Institute of Biotechnology (Shanghai, China). U0126-EtOH was purchased from Apex Bio (Houston, TX, USA). These agents were dissolved in dimethyl sulfoxide (DMSO) to obtain stock solutions and the final working concentration of DMSO was <0.1% (v/v). Antibodies were purchased from the following companies: anti-phospho-Drp1 (#4494), anti-phospho-MAPK Substrates Motif [PXpTP] (#14378), anti-Bax (#2772) and anti-HK-II (#2867) from Cell Signaling Technology (Beverly, MA, USA); anti-Drp1 (ab184247), anti-prohibitin (ab75771), anti-PKC-delta (ab182126) and anti-β-Actin (ab8226) and anti-PKC-epsilon from Abcam (Cambridge, MA, USA); anti-MFF (sc-32577) from Santa Cruz Biotechnology, Inc. (Santa Cruz, CA, USA); anti-GPR91 (BS2961), Goat Anti-Rabbit IgG (H+L), HRP (BS13278), anti-GAPDH (AP0063) and Goat Anti-Mouse IgG (H+L) (Alexa Fluor 488) (BS12478) from Bioworld Technology (St. Paul, MN, USA); Alexa Fluor 647 AffiniPure Donkey Anti-Mouse IgG (H+L) antibody from Yeasen (Shanghai, China); anti-ATP1A1 (Na^+^/K^+^-ATPase 1) (14418-1-AP) and anti-ATP5A1 (66037-1-lg) from Proteintech Group (Manchester, UK); anti-ERK1/2 (CY5487) and anti-phospho-ERK1 (T202/Y204)+ERK2 (T185/Y187) (CY5277) from Abways Technology (Shanghai, China).

### Animals

Neonatal rats (1 or 2 days old) and ICR male mice (18–22 g) were purchased from the Laboratory Animal Center of Nanjing Qinglongshan. The care and treatment of animals were consistent with the Animal Ethics Committee of China Pharmaceutical University.

### Cell preparation and culture

Neonatal rat ventricular myocytes (NRVMs) were prepared as previously described^18^. Briefly, NRVMs were isolated from 1–2-day-old Sprague-Dawley rat, digested with 0.08% collagenase and purified by differential adhesion method. Myocytes were incubated overnight in Dulbecco's modified Eagle's medium (DMEM) containing 10% (v/v) fetal bovine serum (FBS) and 0.1 mmol/L 5-bromo-2-deoxyuridine at 37 °C in a humidified incubator of 5% CO_2_ atmosphere. For oxygen–glucose deprivation (OGD) insult, the NRVMs were exposed to 1% O_2_ with glucose deprivation for 4 h. For succinate treatment, NRVMs were incubated with sodium succinate (200 μmol/L) for 2 h.

H9c2 cells (American Type Culture Collection, Manassas, VA, USA) were maintained in DMEM containing 10% FBS, supplemented with antibiotics (100 U/mL penicillin G and 100 μg/mL streptomycin sulfate), at 37 °C in a humidified atmosphere of 5% CO_2_.

### Isoproterenol-induced myocardial ischemic injury in mice

Mice were treated with intraperitoneal injection of dimethyl malonate (160 mg/kg) or saline 1 h before isoproterenol injection (120 mg/kg, subcutaneous) for 2 consecutive days. At 24 h after the last injection, mice were killed for collection of hearts. For western blot analysis, left ventricle regions were lysed in ice-cold radioimmunoprecipitation assay (RIPA) buffer to extract protein. For immuofluorescent assay and apoptosis assay, the whole heart was directly fixed in 4% paraformaldehyde or 15% sucrose. The infarct size of the hearts was viewed by 2,3,5-triphenyltetrazolium chloride (TTC) staining.

### Measurement of intracellular Ca^2+^ concentration

Intracellular Ca^2+^ was monitored with a Ca^2+^ assay kit. NRVMs were lysed with deionized water followed by 3 times of freezing and thawing cycle. After centrifuging at 10,000 rpm for 10 min at 4 °C, the supernatant was collected for the determination of intracellular Ca^2+^ contents according to the manufacturer’s instructions (Jiancheng Bioengineering Institute).

### Detection of reactive oxygen species (ROS)

After stimulation with indicated agents, NRVMs were loaded with 5 μM DHE working solution (Beyotime Institute of Biotechnology) at 37 °C for 30 min. After washing, cells were fixed in 4% paraformaldehyde for 5 min at 4 °C, and ROS production was measured by a microplate reader.

### Measurement of succinate concentration

After stimulation, NRVMs (1 × 10^6^) or cardiac tissue (10 mg) were rapidly homogenized on ice in 100 μL of succinate assay buffer. Then, the homogenate was centrifuged at 10,000 × *g* for 5 min to collect the supernatant. Intracellular succinate was detected using a Succinate Colorimetric Assay Kit (Sigma-Aldrich).

For measurement of extracellular succinate, NRVMs were treated with indicated agents, and then the conditional medium was collected for the assay according to the manufacturer’s instructions.

### Membrane/cytosol fractionation

Membrane and cytosolic fractions were prepared from NRVMs or cardiac tissues with Membrane and Cytosol Protein Extraction Kit (Beyotime Institute of Biotechnology) referring to the instructions. The samples were moderately homogenized and then centrifuged at low speed to abandon nucleus and intact cells. The supernatants were centrifuged at 14,000 × *g* for 30 min at 4 °C to obtain membrane fraction. Membrane proteins were obtained with membrane protein extraction reagent. The remaining supernatant was taken as the cytosol fraction.

### Cytosol/mitochondria fractionation

Cytosolic and mitochondrial fractions were prepared from NRVMs using Cell Mitochondria Isolation Kit (Beyotime Institute of Biotechnology) according to the manufacturer’s protocol. Briefly, cells were collected and homogenized with a glass homogenizer in mitochondrial isolation buffer on ice. Unlysed cells and nuclei were pelleted by spinning for 10 min at 600 × *g*. The supernatants were centrifuged at 11,000 × *g* for 10 min at 4 °C to obtain mitochondrial pellets. The remaining supernatant was taken as the cytosol fraction. Then, the mitochondrial pellets were lysed to extract mitochondrial protein using lysis buffer.

### Western blot analysis and immunoprecipitation (IP)

Cells were lysed in ice-cold RIPA buffer to obtain protein by centrifugation at 12,000 × *g* for 20 min at 4 °C. Protein concentration was quantified using Enhanced BCA Protein Assay Kit (Beyotime Institute of Biotechnology, Shanghai, China). Equal amount of protein was separated by sodium dodecyl sulfate–polyacrylamide gel electrophoresis (SDS-PAGE), transferred onto polyvinylidene difluoride membranes and then blocked at room temperature for 2 h. The membranes were immunoblotted with primary antibodies at 4 °C overnight. After washing, blots were incubated with horseradish peroxidase (HRP)-conjugated secondary antibody at room temperature for 2 h. Protein bands were visualized with chemiluminescence reagent and quantified by Image-ProPlus 6.0 software.

For immunoprecipitation, collected cells or cardiac tissues were incubated in the ice-cold cell lysis buffer for western and IP (Beyotime Institute of Biotechnology) for 30 min. Cell lysates were then centrifuged at 12,000 × *g* for 20 min and the soluble fraction was collected. Next, anti-Drp1 antibody or anti-MFF antibody was immunoprecipitated overnight at 4 °C and then with protein A+G agarose beads (Beyotime Institute of Biotechnology) for another 2 h. After rinsing with the lysis buffer or phosphate-buffered saline, the beads complexes were then boiled in 1% SDS loading buffer for western blot analysis with the indicated antibodies.

### Transfection of small interfering RNA

To specifically suppress GPR91 expression, H9c2 cells of 60-80% confluence were transfected with small interfering RNA (siRNA) duplex specific for rat GPR91 (sc-270636, Santa Cruz Biotechnology, Santa Cruz, CA, USA) or control siRNA (sc-37007) by siRNA transfection reagent (sc-29528), according to the manufacturer’s instructions. For silencing of PKCδ or MFF expression, H9c2 cells were transfected with rat PKCδ siRNA or rat MFF siRNA (Genomeditech) by Lipofectamine 2000 Reagent (11668-019, Thermo Fisher Scientific, San Jose, CA, USA), using control siRNA (Genomeditech) as the control. After 48 h post transfection, the cells were treated with sodium succinate for 2 h and then collected and processed for immunofluorescence microscopy and western blot.

### Immunofluorescence

After treatment, NRVMs were stained with 200 nmol/L Mito Tracker Red CMXRos (Molecular Probes, Thermo Fisher Scientific, San Jose, CA, USA) to label mitochondria. NRVMs were then fixed in 4% paraformaldehyde for 20 min, and permeabilized with 0.2% Triton X-100 for 10 min at room temperature. After blocking with 3% bovine serum albumin, specimens were then labeled with specific primary antibodies overnight at 4 °C in a humidified chamber, followed by incubation with fluorescent secondary antibodies for 1 h at 37 °C. After washing, specimens were examined with a confocal scanning microscope (Zeiss LSM 700).

### Mitochondrial fission analysis

NRVMs were stained with 200 nmol/L Mito Tracker Red CMXRos for 30 min at 37 °C. The structure of mitochondria was viewed using confocal microscopy (Zeiss LSM 700). Mitochondria with a predominantly intact network of tubular were identified as normal. Cells with disrupted and predominantly spherical mitochondria were identified as having mitochondrial fission.

To detect mitochondrial morphology in heart, cardiac tissues were fixed with 4% paraformaldehyde, dehydrated and embedded in Tissue-Tek O.C.T. Compound (Sakura Finetek, Torrance, CA, USA) and then cut into slices. The slices were stained with ATP5A1 antibody followed by secondary antibody. Mitochondrial morphology was visualized by confocal microscopy (Zeiss LSM 700).

### Measurement of mitochondrial membrane potential

Treated NRVMs were loaded with the potentiometric dye TMRE (500 nmol/L) for 30 min at 37 °C. Fluorescence intensities were determined at the single-cell level using confocal microscopy (Zeiss LSM 700).

### ELISA assay of cytochrome *C*

NRVMs were stimulated with sodium succinate (200 μmol/L) with or without mdivi-1 (10 μmol/L), or treated with hypoxia (1% O_2_) in the presence or absence of dimethyl malonate (5 mmol/L). Cell cytosolic and mitochondrial fractions were collected and the concentration of cytochrome *C* was assayed with commercial enzyme-linked immunosorbent assay (ELISA) kits (R&D, USA).

### Apoptosis analysis

Cell death in the myocardium was assayed in heart paraffin section with an in situ Apoptosis Detection Kit based on the terminal deoxynucleotidyl transferase-mediated dUTP nick end-labeling (TUNEL) system (Roche Applied Science, Upper Bavaria, Germany). The staining was detected by NanoZoomer 2.0-RS (Hamamatsu, Japan). In addition, cell apoptosis in NRVMs was detected with TUNEL assays kit (Vazyme Biotech Co., Nanjing, China) by a confocal scanning microscope (Zeiss LSM 700).

### Statistical analysis

Data are presented as mean values ± SD. The significance of differences was analyzed by one-way analysis of variance followed by the Tukey's test. A value of *p* < 0.05 is considered statistically significant.

## Results

### Succinate stimulation activated GPR91 receptor

**Fig. 1:**
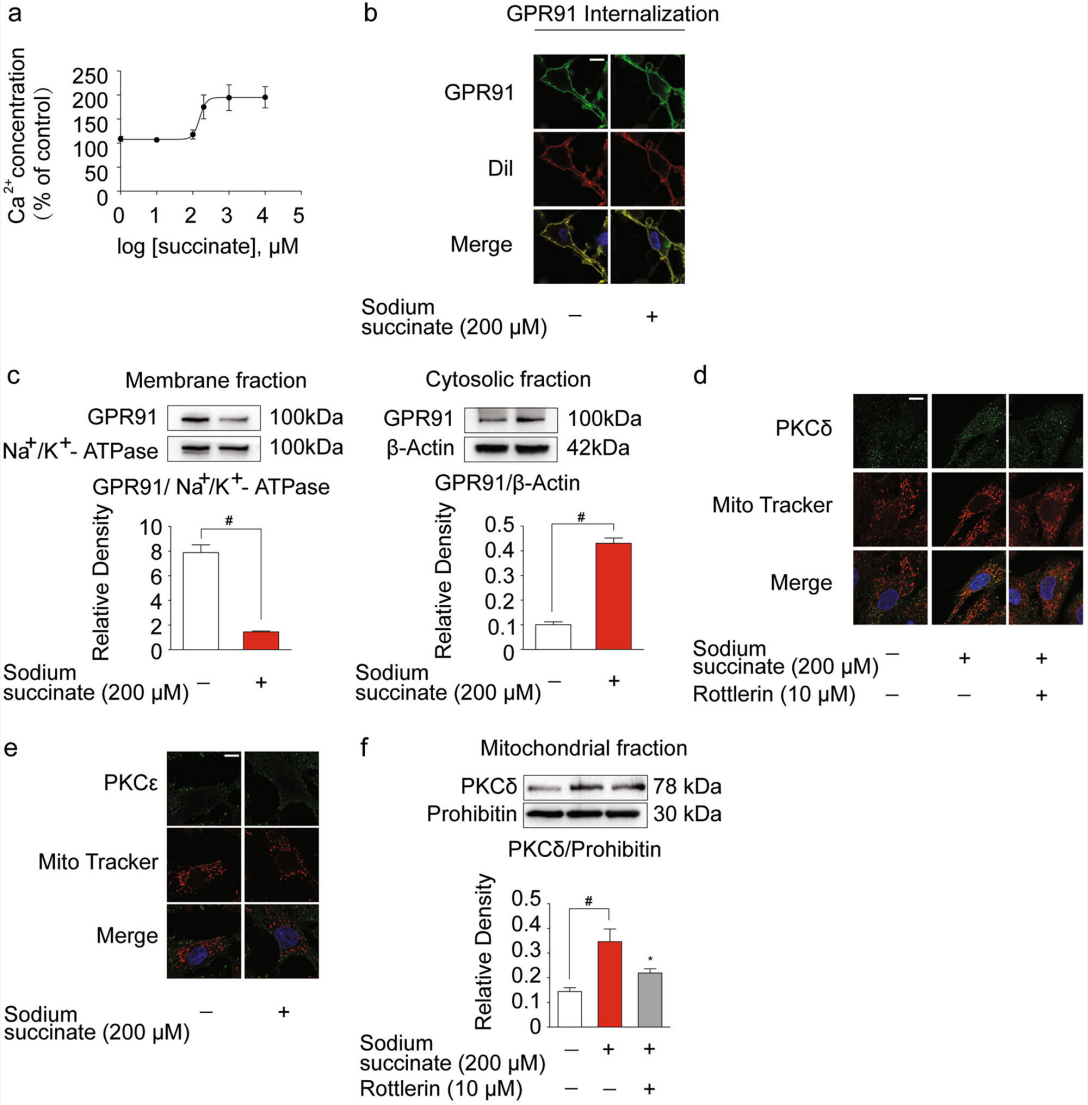
Succinate stimulation activated GPR91 receptor. Neonatal rat ventricular myocytes (NRVMs) were stimulated with sodium succinate for 2 h. **a** Dose response curve of intracellular Ca^2+^ concentration in response to succinate was performed. **b** GPR91 localization was determined by immunofluorescence. Cell membrane was visualized by Cell Tracker CM-Dil. Scale bar: 10 μm. **c** GPR91 expression on cell membrane and in cytosol was determined by western blot. **d**, **e** PKCδ (**d**) or PKCε (**e**) translocation to mitochondria was detected by immunofluorescence. Mitochondria were visualized by Mito Tracker Red CMXRos. Scale bar: 10 μm. **f** Mitochondrial PKCδ expression was explored by western blot. Data are shown as mean ± SD of five independent experiments; *p *<* 0.05 vs sodium succinate-only treatment; ^#^*p* *<* 0.05 vs indicated treatment

It is demonstrated that an increase in intracellular Ca^2+^ concentration is the feature of GPR91 activation^19^. We observed that sodium succinate effectively increased intracellular Ca^2+^ contents at concentrations ranging from 100 μM to 1 mM with half-maximal effective concentration (EC_50_) of 153.9 μM (95% confidence interval, 92.37–256.5 μM) (Fig. 1a). Thus, sodium succinate was used at a working concentration of 200 μM as extracellular succinate to stimulate cardiomyocytes in subsequent experiments, consistent with the previous studies^10,20^. Immunofluorescent staining revealed that GPR91 was localized to the plasma membrane in cardiomyocytes under basal condition. Sodium succinate was capable of inducing GPR91 internalization (Fig. 1b), further confirming the activation of GPR91. Western blot results showed that succinate increased GPR91 expression in the cytosolic fraction, in parallel with decreased expression in membrane (Fig. 1c). PKC activation is a branch of cellular signaling in response to GPR91 activation^13^. Confocal microscopy results showed that succinate induced PKCδ, but not PKCε, translocation to mitochondria (Fig. 1d, e). Correspondingly, succinate increased PKCδ protein expression in mitochondria (Fig. 1f). PKCδ inhibitor rottlerin prevented PKCδ translocation to mitochondria. These results indicated that extracellular succinate activated membrane GPR91 with PKCδ translocation to mitochondria.

### Succinate promoted Drp1 recruitment to mitochondria via GPR91-dependent PKCδ activation

**Fig. 2:**
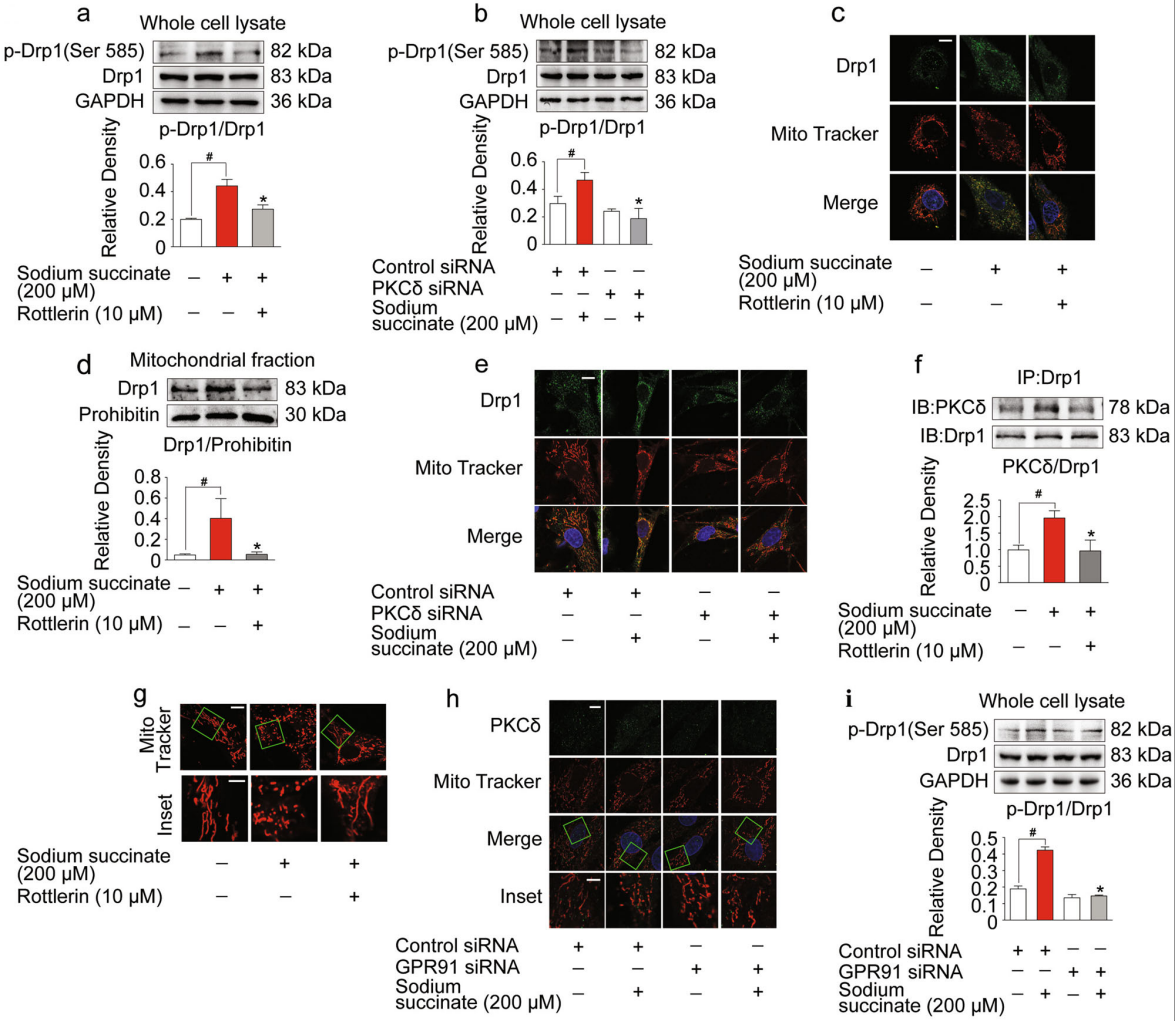
Succinate promoted Drp1 recruitment to mitochondria by GPR91-dependent PKCδ activation. **a**–**g** Neonatal rat ventricular myocytes (NRVMs) were pretreated with or without rottlerin upon sodium succinate stimulation for 2 h. **a** Increased Drp1 phosphorylation (Ser 585) expression in whole cell detected by western blot. **b** Drp1 phosphorylation (Ser 585) in H9c2 cells transfected with PKCδ siRNA. **c** Views of Drp1 recruitment to mitochondria with confocal microscope. Scale bar: 10 μm. **d** Mitochondrial Drp1 expression detected by western blot. **e** Recruitment of Drp1 to mitochondria in H9c2 cells transfected with PKCδ siRNA. Bar: 10 μm. **f** PKCδ/Drp1 interaction determined by co-immunoprecipitation and western blot. **g** Representative confocal images of mitochondrial morphology (upper, scale bar: 10 μm; lower, magnified images, bar: 5 μm). **h** Confocal images of PKCδ translocation to mitochondria (upper, scale bar: 10 μm; lower, magnified images, bar: 5 μm) in H9c2 cells transfected with GPR91 specific or control siRNAs. **i** Western blot of Drp1 phosphorylation (Ser 585) expression in H9c2 cells transfected with GPR91 specific or control siRNAs. Data are shown as mean ± SD of five independent experiments; *p *<* 0.05 vs sodium succinate-only treatment; ^#^*p* *<* 0.05 vs indicated treatment

Mitochondrial dynamics is required to preserve mitochondrial function. Phosphorylation of Drp1 at Ser 585 in rat (equivalent to Ser 616 of human Drp1 isoform 1) promotes Drp1 translocation to mitochondria and induces mitochondrial fission^21,22^. Sodium succinate increased Drp1 phosphorylation (Ser 585) (Fig. 2a) and promoted Drp1 translocation to mitochondria (Fig. 2c, d), whereas these alterations were reversed by PKCδ inhibitor rottlerin and PKCδ knockdown with siRNA treatment (Fig. 2a–e). To determine whether or not Drp1 and PKCδ interacted directly, we isolated Drp1 protein from the cell lysate by immunoprecipitation. Increased PKCδ protein expression was observed in Drp1 protein (Fig. 2f). These results indicated that PKCδ could bind to Drp1 in the cytosol, leading to Drp1 activation. As expected, succinate promoted Drp1 recruitment and caused mitochondrial fragmentation, a morphologic change referred as mitochondrial fission (Fig. 2g). To prove the role of GPR91, we treated H9c2 cells with GPR91 siRNA, and observed that GPR91 knockdown diminished succinate-induced PKCδ translocation to mitochondria and Drp1 phosphorylation (Ser 585) (Fig. 2h, i).

### Succinate activated MFF through ERK1/2 in a GPR91-dependent manner

**Fig. 3:**
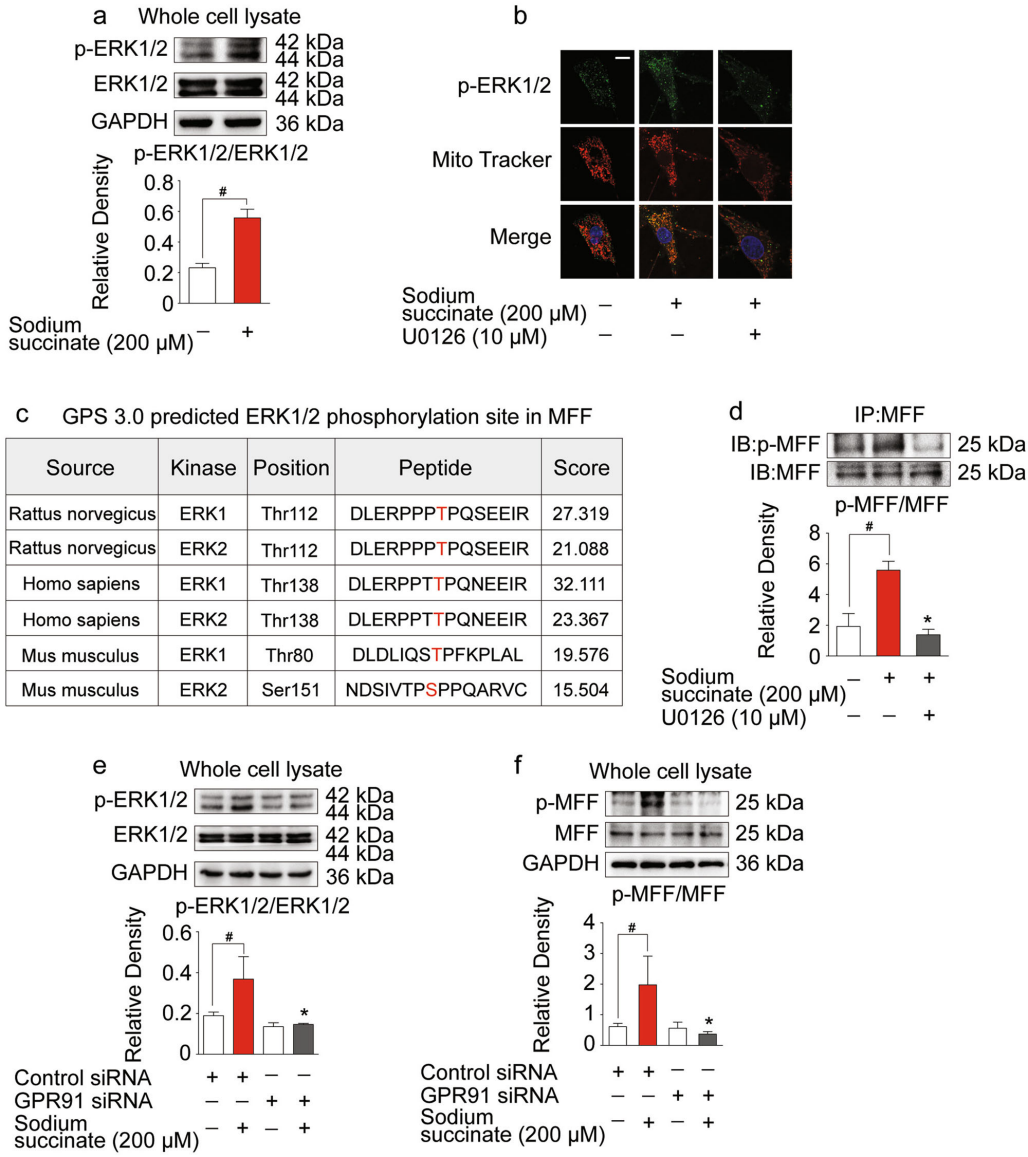
Succinate activated mitochondrial fission factor (MFF) through ERK1/2 in a GPR91-dependent manner. **a** Increased p-ERK1/2 in response to sodium succinate determined by western blot. **b** Confocal images of p-ERK1/2 in mitochondria in the absence or presence of U0126 with sodium succinate stimulation for 2 h. Scale bar: 10 μm. **c** ERK1/2 phosphorylation site in MFF predicted by GPS 3.0. **d** Co-immunoprecipitation and western blot of MFF phosphorylation. **e**, **f** Immunoblot of p-ERK1/2 and p-MFF expression in whole cell lysates when GPR91 is silenced with siRNA with or without sodium succinate. Data are shown as mean ± SD of five independent experiments; *p *<* 0.05 vs sodium succinate-only treatment; ^#^*p* *<* 0.05 vs indicated treatment

Apart from PKCδ activation, ERK1/2 is another signaling branch from membrane GPR91 activation^13,14^. In cardiomyocytes, sodium succinate insult increased ERK1/2 phosphorylation and promoted its translocation to mitochondria (Fig. 3a, b). MFF is a receptor for Drp1 to facilitate mitochondrial fission^6^. To identify the putative sites of MFF phosphorylated by ERK1/2, complete amino acid sequences of MFF were scanned by the Scansite databases (phosphorylation sites identification software: Group-based Prediction System, ver 3.0) for ERK1/2 consensus phosphorylation motifs. Search result revealed that rat MFF contains ERK1/2 consensus phosphorylation sites at T112 (Fig. 3c), conforming to the optimal ERK1/2 motif PXSP/PXTP^23^. Immunoprecipitation showed that phosphorylated ERK1/2 consensus sequence was presented in MFF. Succinate treatment increased MFF phosphorylation, and this alteration was decreased by ERK1/2 inhibitor U0126-EtOH (Fig. 3d). To confirm the role of GPR91, we treated H9c2 cells with GPR91 siRNA, and observed that GPR91 knockdown diminished succinate-induced ERK1/2 phosphorylation and MFF phosphorylation (Fig. 3e, f), indicative of the involvement of GPR91 in ERK1/2 and MFF activation.

### Succinate induced mitochondrial fission by GPR91-dependent Drp1 and MFF interaction

**Fig. 4:**
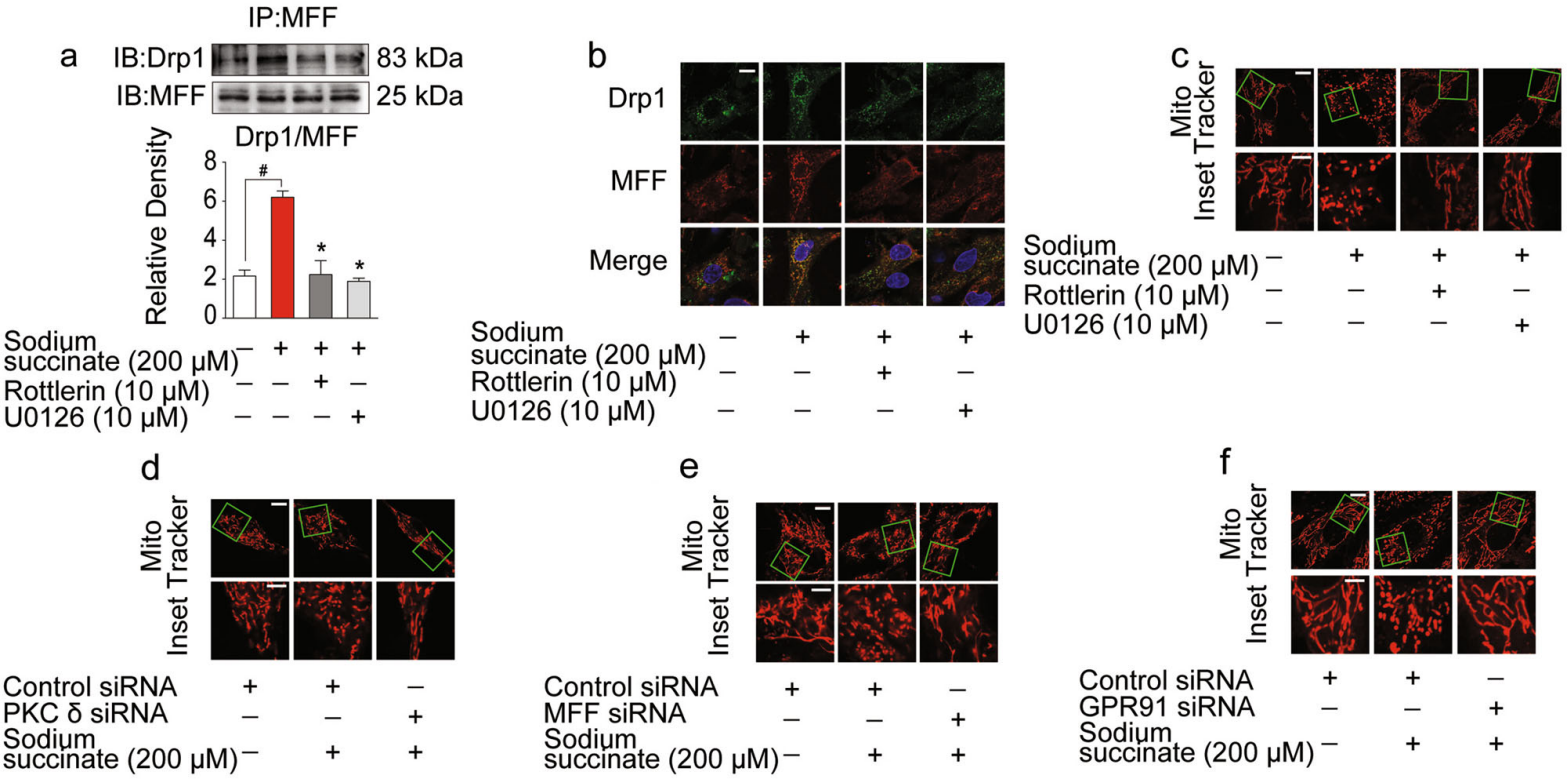
Succinate induced mitochondrial fission by GPR91-dependent Drp1 and MFF interaction. **a**–**c** Neonatal rat ventricular myocytes (NRVMs) were pretreated with or without rottlerin or U0126 upon sodium succinate stimulation for 2 h. **a** The direct combination of Drp1 and MFF was detected by co-immunoprecipitation and western blot. **b** Confocal images of Drp1 and MFF colocalization were observed by immunofluorescent staining. Scale bar: 10 μm. **c** Mitochondrial morphology was visualized using Mito Tracker Red CMXRos (upper, scale bar: 10 μm; lower, magnified images, bar: 5 μm). **d**–**f** Views of mitochondrial morphology in H9c2 cells transfected with PKCδ-, MFF-, and GPR91-specific siRNA respectively (upper, scale bar: 10 μm; lower, magnified images, bar: 5 μm). Data are shown as mean ± SD of five independent experiments; *p *<* 0.05 vs sodium succinate-only treatment; ^#^*p* *<* 0.05 vs indicated treatment

MFF is the primary receptor for Drp1 to facilitate mitochondrial fission. Co-immunoprecipitation showed that succinate increased MFF and Drp1 localization, and this effect was reduced by ERK1/2 inhibitor U0126-EtOH and PKCδ inhibitor rottlerin, respectively (Fig. 4a). Consistently, immunofluorescent staining further revealed that MFF and Drp1 localization increased in response to succinate insult (Fig. 4b). As a result, succinate stimulation induced mitochondrial fission in cardiomyocytes, and this alteration was alleviated by inhibition of PKCδ and ERK1/2 (Fig. 4c). Knocking down PKCδ or MFF prevented mitochondrial fission in response to succinate stimulation (Fig. 4d, e), suggesting the functional importance of PKCδ and MFF. Silencing of GPR91 prevented mitochondrial fission upon succinate treatment, confirming the indispensable role of GPR91 in succinate-induced mitochondrial fission (Fig. 4f).

### Succinate-stimulated mitochondrial fragmentation led to mitochondrial dysfunction and cell death

**Fig. 5:**
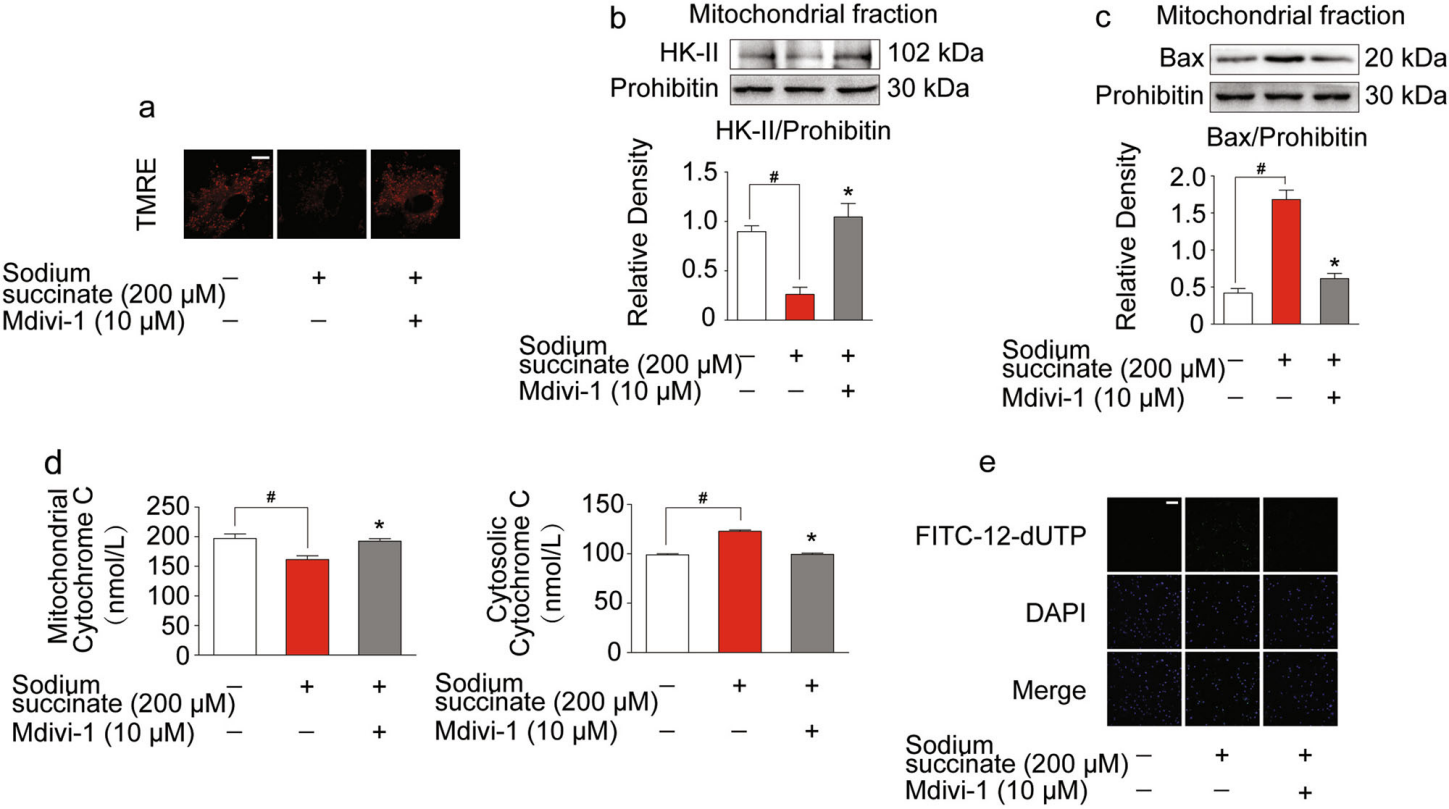
Succinate-stimulated mitochondrial fragmentation led to mitochondrial dysfunction and cell death. Neonatal rat ventricular myocytes (NRVMs) were pretreated with or without mdivi-1 with sodium succinate insult for 2 h. **a** Mitochondrial membrane potential was viewed by tetramethylrhodamine ethyl ester perchlorate (TMRE) labeling with confocal microscopy. Sclae bar: 10 μm. **b**, **c** Immunoblot of mitochondrial HK-II (**b**) and Bax (**c**) by western blot. **d** The levels of cytochrome *C* in mitochondria and cytosol were measured by ELISA. **e** Cell apoptosis analysis was viewed by confocal microscope using TUNEL FITC apoptosis detection kit. Scale bar: 100 μm. Data are shown as mean ± SD of five independent experiments; *p < 0.05 vs sodium succinate-only treatment; ^#^*p* < 0.05 vs indicated treatment

Next, we investigated the functional consequence of succinate-induced mitochondrial integrity impairment. We observed that succinate stimulation induced the collapse of mitochondrial membrane potential, but this action was attenuated by fission inhibitor mdivi-1 (Fig. 5a). Inhibition of mitochondrial fission by mdivi-1 preserved mitochondrial hexokinase II (HK-II) (Fig. 5b). Meanwhile, against succinate insult, mdivi-1 treatment decreased Bax expression in mitochondrial fraction (Fig. 5c). As a result, mitochondrial fission inhibitor mdivi-1 limited cytochrome *C* release from mitochondria, and subsequently reduced cell apoptosis in cardiomyocytes upon succinate insult (Fig. 5d, e). These results demonstrated that succinate impaired mitochondrial integrity and then led to mitochondrial dysfunction and cell death.

### Inhibition of hypoxic succinate release protected cardiomyocytes from OGD injury

**Fig. 6:**
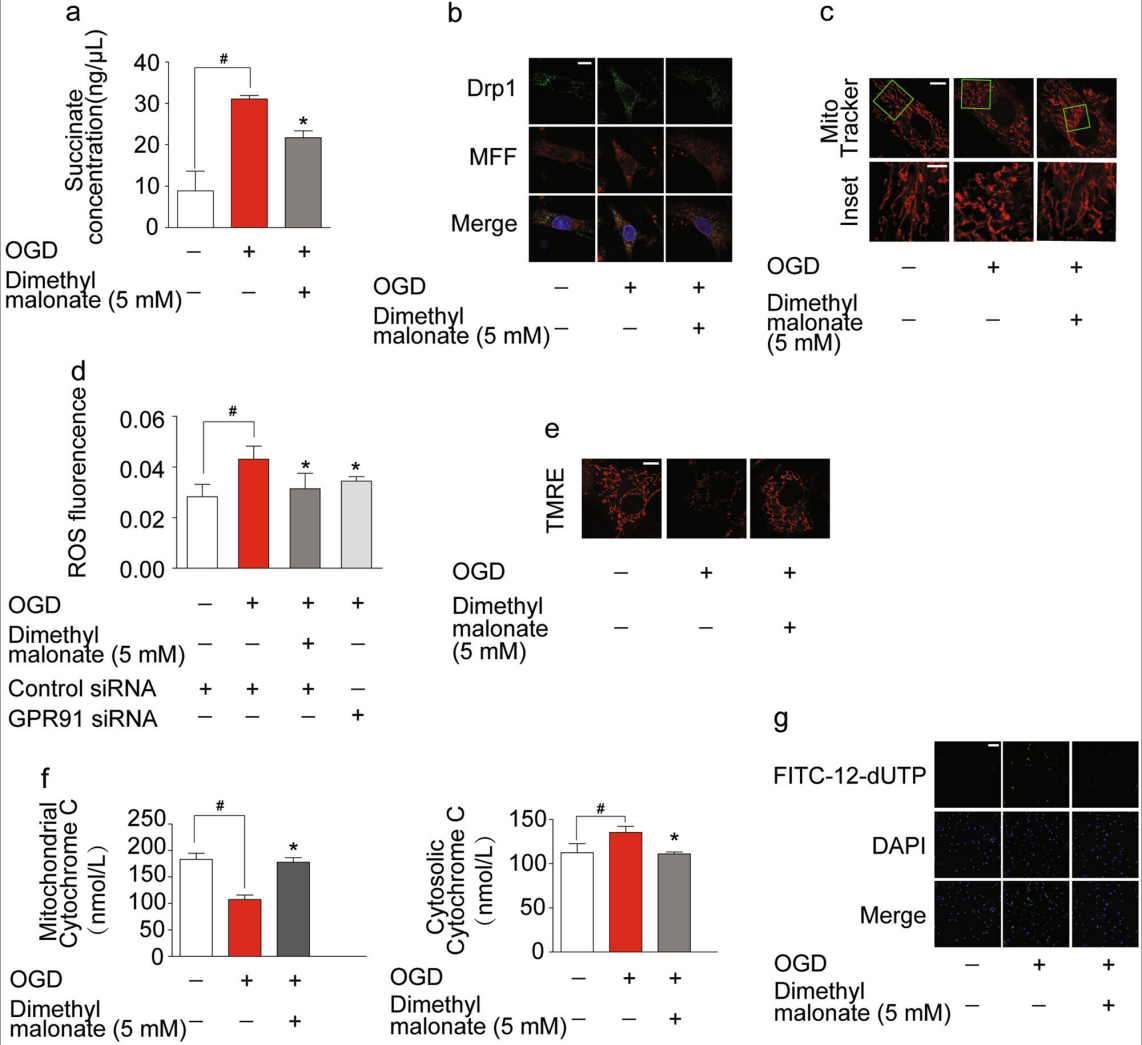
Inhibition of hypoxic succinate release protected cardiomyocytes from oxygen–glucose deprivation (OGD) injury. **a**–**g** Neonatal rat ventricular myocytes (NRVMs) were exposed to 1% oxygen for 4 h in the presence or absence of dimethyl malonate. **a** The level of succinate in condition medium. **b** Views of Drp1 and MFF colocalization observed by immunofluorescent. Scale bar: 10 μm. **c** Mitochondrial morphology was visualized using Mito Tracker Red CMXRos (upper, scale bar: 10 μm; lower, magnified images, bar: 5 μm). **d** ROS production was measured with a fluorescence microplate reader. **e** Mitochondrial membrane potential was visualized with tetramethylrhodamine ethyl ester perchlorate (TMRE) labeling. Sclae bar: 10 μm. **f** The levels of cytochrome *C* in mitochondria and cytosol were measured by an ELISA kit. **g** Cell apoptosis analysis was viewed by confocal microscope using TUNEL FITC apoptosis detection kit. Scale bar: 100 μm. Data are shown as mean ± SD of five independent experiments; *p < 0.05 vs OGD-only treatment; ^#^*p* < 0.05 vs indicated treatment

Hypoxic succinate accumulation is a result from the reversal of succinate dehydrogenase (SDH) activation^24^. SDH inhibitor malonate reduced succinate generation in mitochondria and thereby prevented succinate release during OGD (Fig. 6a). As expected, SDH inhibitor malonate reduced OGD-induced colocalization of MFF and Drp1 (Fig. 6b) and therefore prevented mitochondrial fission (Fig. 6c). Meanwhile, we observed that both malonate and knockdown of GPR91 reduced ROS production under OGD condition, suggesting the possibility that hypoxic succinate release induced oxidative stress with the involvement of GRP91 (Fig. 6d). Malonate pretreatment prevented the loss of mitochondrial membrane potential and reduced mitochondrial cytochrome *C* release, resultantly reducing OGD-induced cell apoptosis (Fig. 6e–g). Although intracellular succinate accumulation could impair mitochondrial function through different mechanisms^20,24^, these results suggested that reduction of cellular succinate accumulation by SDH inhibition contributed to protecting mitochondrial function by limiting succinate release.

### Inhibition of succinate accumulation protected heart from isoproterenol- induced myocardial ischemia injury in mice

**Fig. 7:**
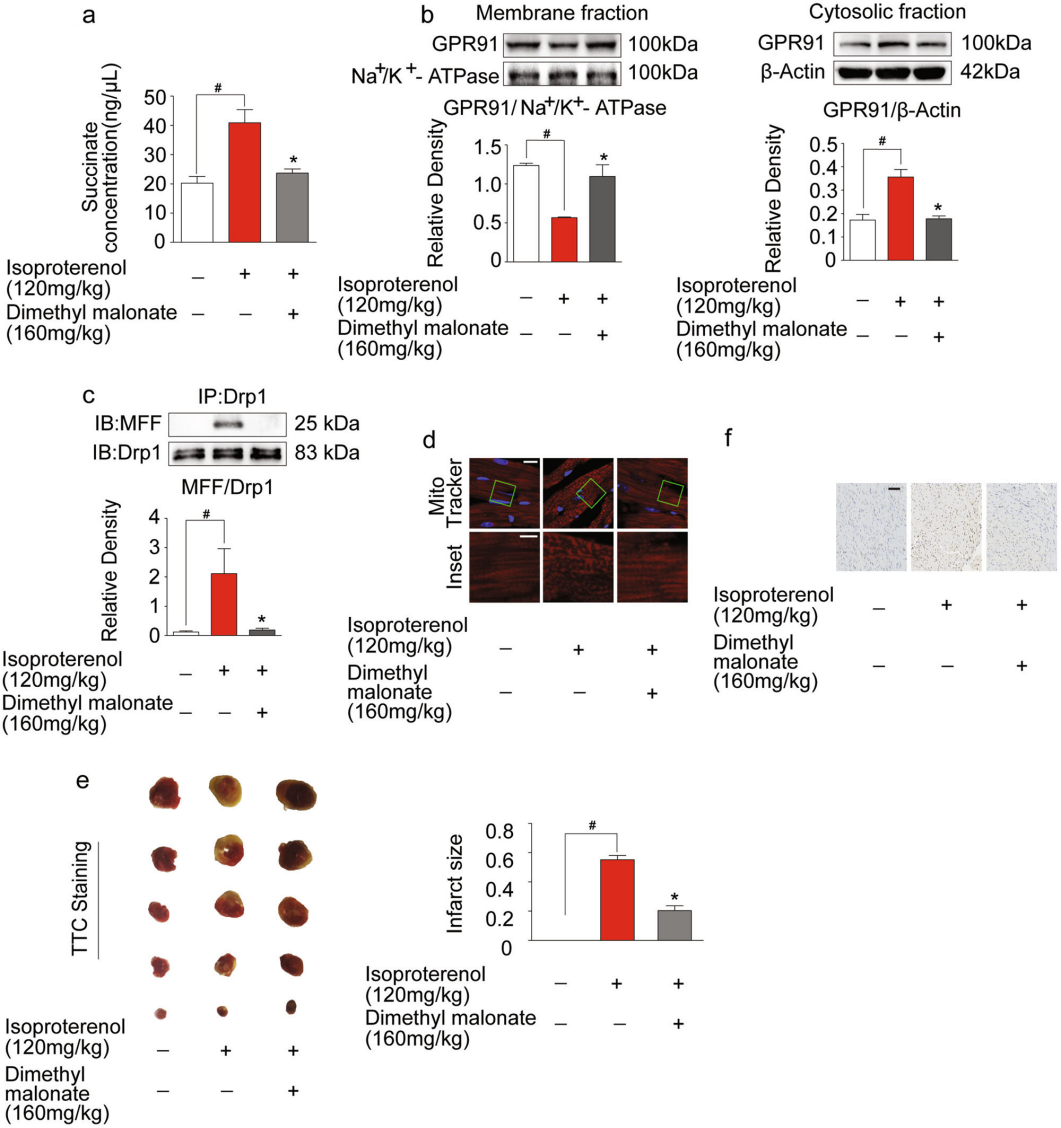
Inhibition of succinate accumulation protected heart from isoproterenol-induced myocardial ischemia injury in mice. **a**–**e** Succinate production (**a**), GPR91 expression in membrane fraction and cytosolic fraction (**b**), Drp1/MFF interaction by co-immunoprecipitation and western blot (**c**), mitochondrial morphology, upper, scale bar: 10 μm; lower, magnified images, bar: 5 μm (**d**), infarct staining with TTC (**e**) and heart paraffin section stained with TUNEL (**f**, scale bar: 100 μm) in isoproterenol-induced myocardial ischemia mice. Data are shown as mean ± SD of six independent experiments; *p < 0.05 vs isoproterenol-only administration; ^#^*p* < 0.05 vs indicated treatment

To test the effects of succinate in vivo, we prepared isoproterenol-induced cardiac ischemia model in mice. We found that succinate accumulated in the ischemic heart, whereas the accumulation was reduced by malonate treatment (Fig. 7a). Malonate administration decreased GPR91 expression in the cytosolic fraction, while the expression in membrane was preserved (Fig. 7b). Because GPR91 activation is special for extracellular succinate^10^, these results suggested that accumulated succinate in mitochondria was released to activate membrane GPR91. Subsequently, malonate treatment attenuated colocalization of MFF and Drp1 in mitochondria and then prevented mitochondrial fission (Fig. 7c, d) in the heart. Moreover, malonate limited infarct size (Fig. 7e) and reduced apoptosis (Fig. 7f) in the heart against isoproterenol-induced ischemic injury.

## Discussion

Altered mitochondrial dynamics and function are considered the initial causes for cardiac injury^3,25^. Intracellular succinate mediates oxidative stress due to reverse electron transport during reperfusion^24^; however, it remains unknown whether extracellular succinate is also implicated in ischemic injury. Herein, we showed that succinate released to extracellular space acted as a signaling molecule to mediate mitochondrial fission via GPR91 signaling pathways in a paracrine and autocrine manner. These findings provided a molecular mechanism to link metabolic dysregulation with impaired mitochondrial integrity.

Apart from being an intermediate in the citric acid cycle, succinate is regarded as a signaling molecule involved in the regulations of cellular responses, including inflammation, hypoxia, oxidative stress and tumorigenesis^26,27^. Succinate is produced within mitochondria, and can translocate into the cytosol through specific transporter SLC25A10^28^, and release to the extracellular space due to local energy metabolism disturbances^12,29,30^. In line with these reports, we found that succinate contents in the culture medium were elevated in response to OGD and isoproterenol challenge in mice induced succinate accumulation in the ischemic heart, suggesting the possibility that the released succinate might influence cellular response in an autocrine/paracrine manner.

GPR91 is a plasma membrane receptor presenting in many tissues, including the heart^17^, and acts as a specific sensor for extracellular succinate^12^. We observed that sodium succinate stimulation activated GPR91 in cardiomyocytes, evidenced by the internalization of the receptor. GPR91 is a Gq-coupled receptor which can mediate the PKC/mitogen-activated protein kinase (MAPK) signaling cascades^12,31^. Mitochondrial morphology is dynamically controlled by Drp1 and the sub-mitochondrial localization of Drp1 is essential for Drp1 to mediate mitochondrial fission. Two sites are phosphorylated on Drp1^1^. Phosphorylation of Drp1 at Ser 616 residue in humans, corresponding to Ser 585 in rats, is able to activate Drp1^21,22^, while Ser 637 phosphorylation can inhibit Drp1 activation^32^. In the present study, upon succinate stimulation, PKCδ combined with Drp1 in the cytoplasm, leading to Drp1 phosphorylation and then translocation of PKCδ with phosphorylated Drp1 to mitochondria to induce mitochondrial fission. Knockdown of GRP91 reduced PKCδ translocation to mitochondria and prevented mitochondrial fission, providing evidence that succinate-mediated GRP91 activation induced mitochondrial fission through regulation of PKCδ. This regulation is consistent with the published study which shows that PKCδ induced aberrant mitochondrial fragmentation in neuronal cells^7^.

Intriguingly, we found that PKCδ but not PKCε is activated in response to GPR91 stimulation. PKCδ and PKCε, the principal members of the novel group of PKCs, are opposed by their principal effects. In the heart, PKCε is favorably involved in the preconditioning protection against ischemic insult, while PKCδ serves as a mediator for the ischemic damage^33^. Our previous study also showed that in response to succinate-mediated GPR91 activation, PKCδ was activated and translocated to mitochondria, impairing pyruvate dehydrogenase activity in cardiomyocytes^20^. Herein, we identified another pathological mechanism by which PKCδ activation impairs mitochondrial integrity in cardiomyocytes. The defects in mitochondrial dynamics can impair energy metabolism, leading to mitochondrial dysfunction^34^. Therefore, we reasoned that PKCδ is involved in both mitochondrial morphological and functional regulation in cardiomyocytes. In fact, PKCδ has multiple protein substrates. By phosphorylating such diverse substrates at different subcellular locations, PKCδ mediates a variety of cellular responses^35^.

Upon activation, Drp1 recruits to mitochondria, where it binds to MFF to assemble fission sites. Evidence shows that phosphorylation of MFF by AMPK may increase the association of Drp1 with mitochondria to induce mitochondrial fission^9^. This evidence indicates that MFF phosphorylation facilitates Drp1 binding to mitochondria to influence the dynamic change of mitochondrial morphology. In cardiomyocytes, we showed that MFF contains ERK1/2 consensus phosphorylation sites, indicative of the potency to be regulated by ERK1/2. Importantly, MAPK/ERK signaling is considered to be a downstream regulation by GRP91 activation^13,14^. Succinate-mediated GPR91 activation increased ERK1/2 phosphorylation and promoted ERK1/2 translocation to mitochondria, where it phosphorylated MFF to facilitate Drp1 localization on mitochondria, contributing to mitochondrial fission. Even though it is documented that ERK can directly phosphorylate Drp1 to promote mitochondrial fission^8,36,37^, our work demonstrated another pathway to regulate Drp1 activation by phosphorylation of MFF. Together with the regulation of PKCδ, our study elucidated that PKC/ERK signaling cascades linking GPR91 activation mediated mitochondrial fission upon extracellular succinate stimulation.

Aberrant mitochondrial fragmentation is the early stages of apoptosis^38^. The structural and functional integrity of mitochondria is essential for mitochondrial HK-II binding which is associated with permeability barrier of inner mitochondrial membrane^39^. Succinate-induced mitochondrial fission resulted in the HK-II dissociation from mitochondria, leading to the collapse of mitochondrial membrane potential. Bax and Drp1 colocalize together at scission sites on mitochondria, enabling cytochrome *C* release from mitochondria^40,41^. We observed that succinate insult increased Bax expression in mitochondrial fraction and promoted cytochrome *C* release from mitochondria, and subsequently induced cell apoptosis in cardiomyocytes. These alterations were reversed by mitochondrial fission inhibitor mdivi-1, demonstrating that mitochondrial fission contributed to succinate-mediated mitochondrial dysfunction and cell death.

The hypoxic succinate accumulation within cells is a universal metabolic signal in heart^42^, mediating oxidative stress^43^. A recent study shows that accumulated intracellular succinate acts as a potential electron store that fuels to drive ROS formation from mitochondrial complex I by reverse electron transport during reperfusion^24^. Differently, the present work suggested that under hypoxic conditions, excessive extracellular succinate could induce ROS production in response to GPR91-induced mitochondrial fission, which may contribute to exaggerating ROS production to induce oxidative damage during subsequent reperfusion.

To translate our findings in vivo, we observed the protective effects of SDH inhibitor malonate in isoproterenol-induced cardiac ischemia model. We observed that succinate substantially accumulated in the heart and induced GPR91 activation. As expected, SDH inhibitor malonate reduced succinate accumulation in the heart and decreased GPR91 activation against ischemic insult, a regulation likely due to the limited succinate release. Consequently, malonate blocked MFF and Drp1 colocalization in mitochondria and prevented mitochondrial fission. The attenuated heart injury by malonate treatment provided in vivo evidence to support the conclusion that inhibiting succinate-mediated GPR91 signaling may protect the heart from ischemic injury, at least in part.

**Fig. 8:**
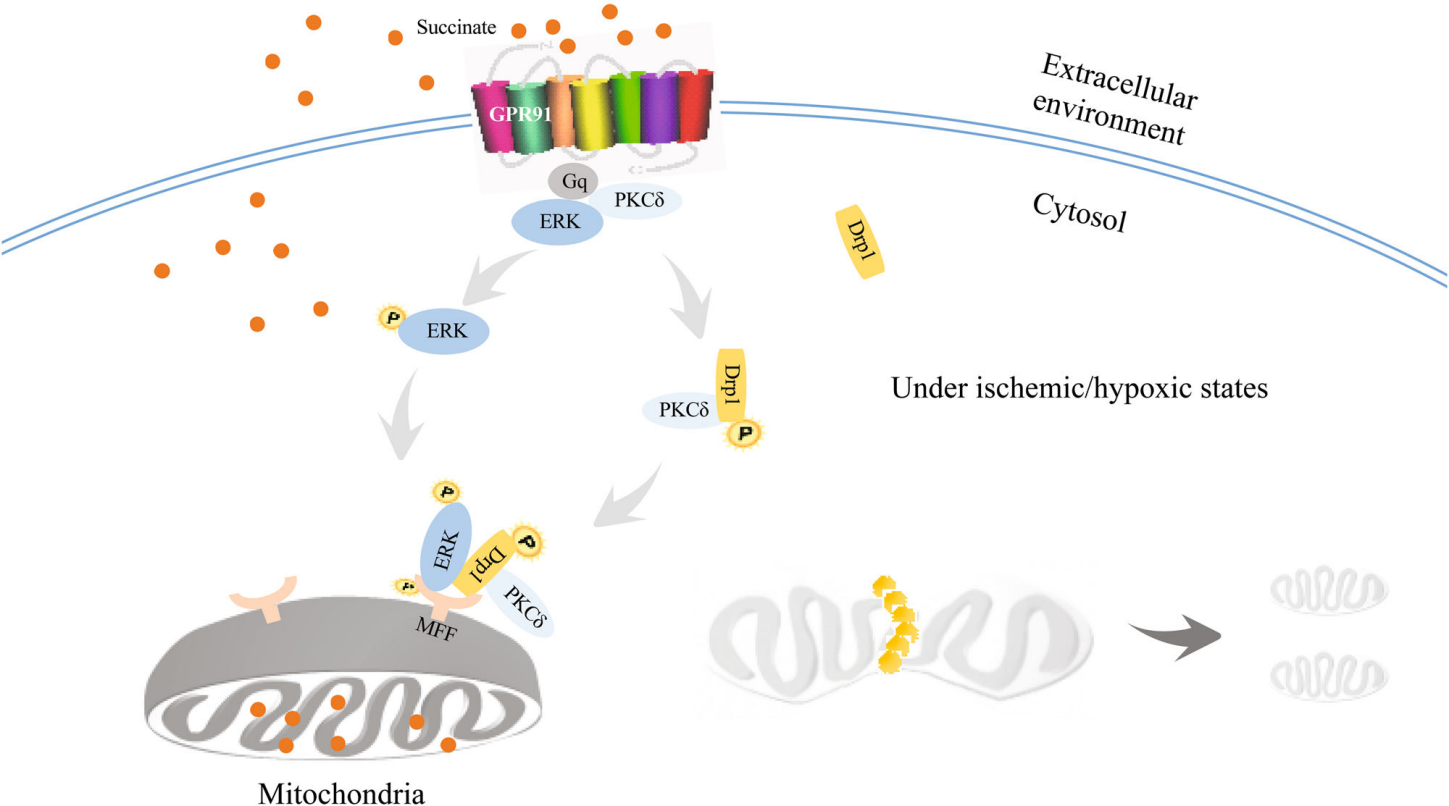
The proposed pathway through which extracellular succinate induced mitochondrial fission in a GPR91-dependent manner in cardiomyocytes. Under hypoxic and ischemic conditions, accumulated succinate was released to extracellular milieu. Extracellular succinate activated GPR91 receptor and its downstream PKCδ/ERK1/2 signaling cascades. Succinate increased the translocation of Drp1 to mitochondria via PKCδ activation, and induced MFF phosphorylation via ERK1/2 activation both in a GPR91-dependent manner, leading to mitochondrial fission

Taken together, we showed that hypoxic succinate accumulation and release could activate membrane GPR91 and induce mitochondrial fission through PKCδ/ERK1/2 signaling pathways (Fig. 8). These results suggested that inhibition of succinate-mediated GPR91 activation might be a potential therapeutic strategy for protecting cardiomyocytes from ischemic injury.
